# Clinical features and computed tomography findings of retrobulbar disease in cats

**DOI:** 10.3389/fvets.2025.1743613

**Published:** 2026-01-12

**Authors:** Kristen Hasegawa, Anna Vincek, Janny V. Evenhuis, Stephanie Goldschmidt, Maria Soltero-Rivera, Natalia Vapniarsky, Kathryn Good, Claudio J. Gutierrez, Boaz Arzi

**Affiliations:** 1 William R. Pritchard Veterinary Medical Teaching Hospital, School of Veterinary Medicine, University of California, Davis, Davis, CA, United States; 2 Department of Surgical and Radiological Sciences, University of California, Davis, Davis, CA, United States; 3 Department of Pathology, Microbiology and Immunology, School of Veterinary Medicine, University of California, Davis, Davis, CA, United States; 4 Department of Anatomy, Physiology and Cell Biology, School of Veterinary Medicine, University of California, Davis, Davis, CA, United States

**Keywords:** exophthalmos, feline, infection, inflammation, neoplasia, orbit, pain, retropulsion

## Abstract

The objective of this study was to describe the clinical features and computed tomography (CT) findings of cats affected with retrobulbar disease. The medical records of 37 client-owned cats diagnosed with retrobulbar disease between the years 2009–2024 were reviewed. Clinical information, signalment, the presenting specialty service, clinical signs, diagnostic results, treatment, and outcomes were documented. CT features of retrobulbar disease as well as cytology and histopathology were reviewed. Fifteen cats (40.5%) were diagnosed with primary disease in the retrobulbar space and 22 cats (59.5%) were diagnosed with secondary retrobulbar disease. Out of the 15 cats with primary retrobulbar disease, 9 were diagnosed with neoplasia, 3 were diagnosed with an infectious/inflammatory process, 2 were traumatic in origin, and 1 cat had a cyst. Of the 22 cats with secondary retrobulbar disease, 21 cats were diagnosed with neoplasia and 1 cat was diagnosed with an infectious disease. CT findings of orbital osteolysis and reduction of retrobulbar fat were significantly associated with neoplasia. Survival outcomes for cats diagnosed with a primary retrobulbar infectious/inflammatory disease were significantly better than for those diagnosed with primary or secondary retrobulbar neoplasia. This study found that neoplasia is the most common primary and secondary retrobulbar disease in cats. Due to the significantly different prognostic implications between cats with primary infectious/inflammatory retrobulbar disease, primary neoplasia and secondary neoplasia, we also confirmed that CT is an essential part of diagnosis and characterization of the extent of the disease, and that additional diagnostics such as histopathology, cytology, culture and susceptibility, or fungal cultures are needed to further support and guide treatment options. Finally, cats that present with either primary infectious/inflammatory or traumatic retrobulbar disease carry favorable prognosis with either medical or surgical intervention.

## 1 Introduction

**Figure 1:**
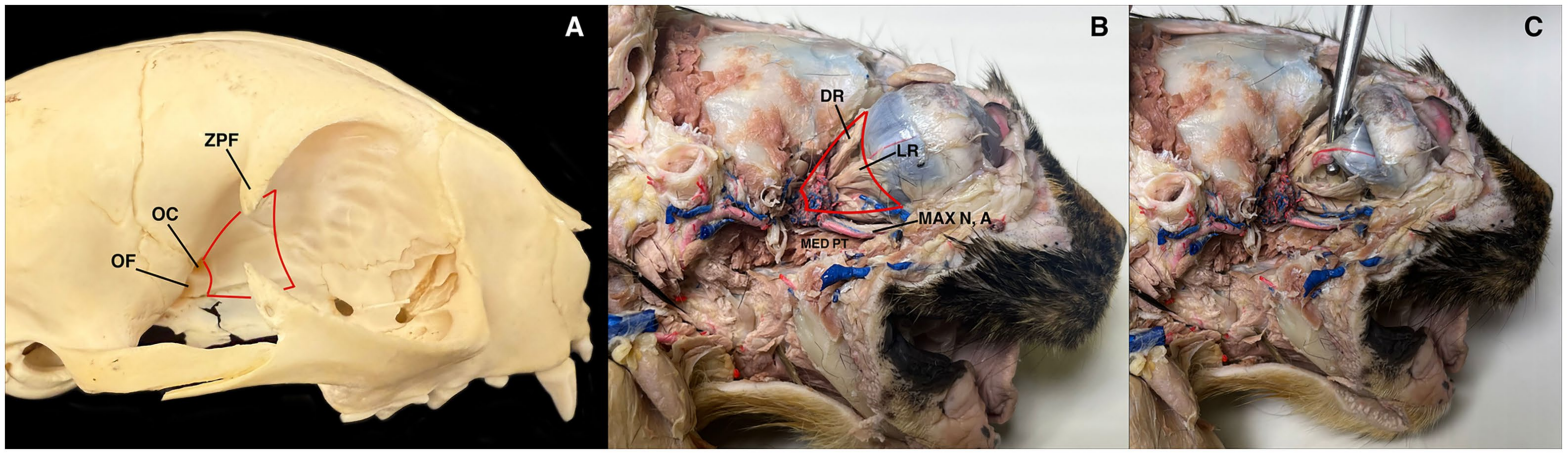
**(A)** The retrobulbar space in a cat (red lines). ZPF, zygomatic process of the frontal bone; OC, optic canal; OF, orbital fissure. **(B)** Retrobulbar scape in a cat (red lines). The eye was injected with distilled water. MAX N, A, Maxillary nerve and artery; MED PT, medial pterygoid muscle (transected); DR, dorsal rectus muscle; LR, lateral rectus muscle. **(C)** The probe is holding the optic nerve and a branch of the internal ophthalmic artery. In the dissection images, the zygomatic arch and the ramus of the mandible have been removed.

The retrobulbar space is an anatomically complex region. Disease in this location typically results in significant morbidity and mortality (1). The orbit is composed of a cavity that is enclosed by the orbital bones, fascia, extraocular muscles, adipose tissue, and the eyeball. The retrobulbar space is located adjacent to the eyeball and contains tissues including the zygomatic gland, extraocular muscles, nerves, blood vessels and muscles of mastication (in the orbital floor) (Figure 1). The nasal cavity, paranasal sinuses, cranial cavity, and oral cavity are closely associated with the retrobulbar space (2). In dogs affected by retrobulbar disease due to inflammatory conditions, the prognosis is typically good with 70% of patients making full recovery (3). However, ocular complications are common, highlighting the importance of multidisciplinary collaboration to optimize treatment. The prevalence of permanent visual impairment is approximately 12% in dogs and is associated with suspected optic nerve compression, vascular injury and vasculitis or inflammatory vs. infiltrative optic neuropathy (3). Primary retrobulbar disease includes neoplasia, inflammatory/infectious, or traumatic disease processes that originate in the retrobulbar space. Secondary retrobulbar disease is defined as the neoplasia or inflammatory/infectious process that originates elsewhere and infiltrates the retrobulbar space (1, 2).

Orbital and retrobulbar neoplasia is reported to be mostly malignant in both dogs and cats (1, 2, 4, 5). Squamous cell carcinoma, undifferentiated carcinomas, lymphoma, malignant melanoma, adenocarcinoma, fibrosarcoma, chondrosarcoma, hemangiosarcoma, feline restrictive orbital myofibroblastic sarcoma, unclassified round cell tumors, extramedullary plasmacytomas, meningioma, and angioleiomyomas are previously reported orbital and retrobulbar space neoplasia (1, 2, 4–11). However, the most common type of retrobulbar neoplasia reported in cats is secondary to nasal lymphoma followed by carcinoma (2, 4). Meningioma causing secondary retrobulbar disease has also been reported in dogs but not in cats (8). Previous research reported poor prognosis and survival time of cats diagnosed with retrobulbar neoplasia (2). Non-neoplastic differential diagnoses for orbital and retrobulbar disease in cats include infectious etiologies (e.g., bacterial, fungal, parasitic such as “Onchocerca lupi”), cellulitis, foreign bodies, and trauma (10, 12–21). Clinical signs of orbital and retrobulbar disease typically include pain upon opening the mouth, reluctance to open the mouth, exophthalmos, enophthalmos, and globe deviation (2, 8, 21).

It is recommended that patients with suspected retrobulbar disease pursue advanced diagnostic imaging, diagnostic sampling and targeted treatment. Contrast enhanced computed tomography (CT), magnetic resonance imaging (MRI), and ultrasound are all appropriate diagnostic modalities which carry distinct benefits and pitfalls pending the underlying disease condition. In previous studies, low frequency B-scan ultrasonography was used to aid in the identification of the space-occupying masses in the posterior portions of the eyeball and the orbit (22, 23). Ultrasonography was an adequate imaging modality at diagnosing foreign bodies due to hyperechogenicity and distal acoustic shadowing of a foreign body, as well as surrounding tissue changes (23–25). However, ultrasonography gave both false negative and false positive diagnoses for neoplastic masses as it is reported to misdiagnose large tumors (24–26). Also, the accuracy of diagnosing ocular neoplasia with ultrasonography varies depending on the targeted anatomy (27). MRI is known to be superior to ultrasound as the extent of tumors or abscesses may be identified more clearly (26, 28, 29). Contrast enhanced MRI may also be superior to CT as it can be used to identify intracranial involvement, assess muscle changes associated with inflammation, and outlining abscesses (26). In a previous study, MRI was the only imaging modality that could delineate the border of a suggested retrobulbar neoplasm in a cat compared to skull radiographs, and conventional CT (29). Last, conventional CT with and without contrast is more sensitive to detect skeletal and mineralization changes compared to ultrasound and radiographs (28–31). Orbital bone lysis is one of the most common characteristics observed in CT that can indicate orbital disease in cats (31). A previous study by our group utilizing CT with and without contrast demonstrated the essential role of this imaging modality to diagnose disorders of the retrobulbar space in dogs (1). We also demonstrated that while CT was essential to support a diagnosis, biopsy, cytology, culture and sensitivity, and/or exploratory surgery were necessary to obtain a definitive diagnosis to best direct treatment and discuss prognosis (1). However, similar comprehensive data on the CT findings of retrobulbar disease in cats, and the clinical utility of this imaging modality, is lacking in the literature.

The objective of this study was to describe the clinical features and computed tomography (CT) findings of cats affected by retrobulbar disease. We hypothesized that neoplastic lesions would be more common than inflammatory disease processes in the retrobulbar region of cats. We further hypothesized that retrobulbar mass effect, exophthalmos, and orbital osteolysis will be more commonly associated with neoplastic lesions than inflammatory disease processes, as seen in the dog (1). We also hypothesized that cats diagnosed with infectious/inflammatory retrobulbar disease will have a longer survival time compared to those with neoplasia.

## 2 Materials and methods

### 2.1 Case selection criteria

The electronic medical record database of the University of California, Davis, William R. Pritchard Veterinary Medical Teaching Hospital (UCD-VMTH) was searched to identify cats examined between January 2009 and December 2024 (16 years inclusivity) that were diagnosed with retrobulbar disease. The main inclusion criterion was pathology involving the retrobulbar space evident with conventional CT, which was achieved by searching CT reports with the keyword “retrobulbar.” Cases were included if the etiological diagnosis was confirmed with biopsy/histopathology report, cytology, bacterial or fungal culture, exploratory surgery, necropsy, and/or positive response to antibiotics or anti-inflammatory medications. Cases were excluded if diagnostic results were inconclusive or if diseases were not specifically in the retrobulbar space. There were no specific follow-up criteria necessary for inclusion in this study.

### 2.2 Medical records overview

Medical records were reviewed and the following information was obtained: age at the time of the diagnosis and CT scan, sex, breed, the specialty service that the cat was presented to at the UCD-VMTH, clinical signs observed by the client, physical examination findings, the clinicopathological or histopathologic diagnostic results concluded from the retrobulbar space and the diagnostic modality utilized (i.e., biopsy, fine needle aspiration and cytology, bacterial culture and susceptibility, fungal culture, or necropsy), diagnosis, treatments (if pursued) and outcomes (if known). Cases were categorized as either primary or secondary retrobulbar disease and further separated into either neoplastic, inflammatory/infectious diseases, traumatic, or other (i.e., cyst) as previously described (1).

Clinical signs that were reported by client were reviewed. Clinical signs were divided into the following categories: ocular (exophthalmos, periorbital swelling, chemosis, blepharospasm, and/or ocular discharge, strabismus), respiratory (difficulty breathing, nasal discharge, epistaxis, sneezing, stertorous breathing, facial asymmetry, open mouth breathing), oral (difficulty and/or painful while eating, difficulty opening the mouth), and neurologic (circling, pacing, ataxia). If multiple different clinical signs were reported, then these were placed in a combination category such as both nasal and ocular signs.

Physical examination findings were categorized similarly as above. These clinical signs were recorded potentially relating to the retrobulbar disease and grouped into the following categories: ocular (decreased retropulsion, exophthalmos, ocular discharge, strabismus), respiratory (decreased nasal airflow, epistaxis, nasal discharge – mucoid or serosanguineous), or oral (pain on opening the mouth, evident oral mass or discharge observed).

Treatment was categorized into two general categories: medical or surgical treatment. Medical management was categorized into different types: antibiotics, anti-inflammatory medications, chemotherapy, or radiation therapy with either curative or palliative intent. Surgical management included: orbital exenteration, mass excision, or surgical exploration with abscess drainage.

### 2.3 Radiologic examination

CT reports were initially screened to categorize affected cats for primary or secondary retrobulbar disease. The CT images were then reviewed by a board-certified radiologist (AV) to analyze the cases, to confirm whether the affected cat had primary or secondary retrobulbar disease and to determine a presumptive underlying etiology. CT reports generated during the initial scan were not referenced. New observations and conclusions were made by viewing CT images by the author (AV). The orbital wall and zygomatic arch were evaluated for osseous distortion, osteolysis, or periosteal reaction. Zygomatic salivary gland compression/displacement or enlargement was evaluated (scored subjectively compared to the contralateral, unaffected retrobulbar space). The retrobulbar space was evaluated for reduction of retrobulbar fat or if a mass effect/mass was present (scored subjectively compared to the contralateral, unaffected retrobulbar space). A mass effect was defined as displacement or distortion of the retrobulbar structures in the absence of a definable mass. The globes were evaluated for exophthalmos, enophthalmos, or deformation (scored subjectively compared to the contralateral, unaffected retrobulbar space). The mandibular and retropharyngeal lymph nodes were evaluated for disease (scored subjectively compared to the contralateral, unaffected side). Involvement of other regional bones such as the mandible, maxilla, and nasal sinuses were included.

### 2.4 Histologic examination

Available histopathology slides were reviewed by a board-certified pathologist (NV). Representative photomicrographs are included within this manuscript.

### 2.5 Statistical analysis

The aims of this retrospective study were to categorize retrobulbar disease as either primary or secondary disease processes, and to complete a cross-sectional study in which computed tomography findings of cats with retrobulbar neoplasia vs. infection/inflammation are described and compared. The sample of 37 cats was divided into two groups (i.e., primary or secondary retrobulbar disease) based on the CT findings. These groups were further divided by disease etiology based on diagnostic testing. For this portion of the study, statistical analysis comprised of summary statistics for demographics, clinical signs, physical exam findings, treatments, and outcomes.

Secondly, the sample population was again divided into two groups (neoplastic vs. infectious/inflammatory) based on finalized diagnostic testing performed. Next, CT scan findings of patients of each etiology were compared. Potential differences between neoplastic and infectious/inflammatory retrobulbar disease were assessed by a Pearson’s Chi-square test for independence for each CT finding. All patients were included in calculation of sensitivity and specificity, as all categories or disease may contribute false positive or false negative observations. The results of the Kaplan–Meier survival curve were assessed with a Logrank (Mantel-Cox) test and a Pearson’s Chi-square test.

## 3 Results

Between January 2009 and December 2024, 47 cats underwent CT examination for which a CT report was generated that contained the word “retrobulbar.” All cases that underwent a cone beam computed tomography (CBCT), magnetic resonance imaging (MRI), or a positron emission tomography (PET) scan were excluded from the study. Upon further review of CT images by the authors, six cases were excluded from the study, because the disease process was determined to reflect orbital disease rather than retrobulbar disease. Two cases were excluded due to underlying chronic temporomandibular joint disease rather than true retrobulbar disease. Two additional cases were excluded as they did not have conclusive results. Of the 37 cats that met the inclusion criteria, 15 cases (40.5%) were diagnosed with primary retrobulbar disease, and the remaining 22 cases (59.5%) were diagnosed with secondary retrobulbar disease. The age of cats ranged from 3 months to 18 years, with a mean age of 10 years. The weight of these patients ranged from 0.5 kg to 10 kg. Specialty services to which the cats with retrobulbar disease presented are listed in Table 1.

**Table 1:** The number of cats with primary (P) and secondary (S) retrobulbar pathology that were presented to each specialty service.

	Neoplasia		Infectious/Inflammatory		Trauma		Other	
	P	S	P	S	P	S	P	S
Ophthalmology	6	4	1	0	0	0	0	0
Internal medicine	1	6	1	1	1	0	0	0
Medical oncology	1	4	0	0	0	0	0	0
Radiation oncology	0	5	0	0	0	0	0	0
Dentistry and oral surgery	0	1	1	0	0	0	0	0
Soft tissue surgery	0	1	0	0	0	0	0	0
Emergency	1	0	0	0	1	0	0	0
Neurology	0	0	0	0	0	0	1	0

The rows are organized by specialty service to which the cat was originally presented; the columns are organized by disease etiology.

### 3.1 Primary retrobulbar disease

**Figure 2:**
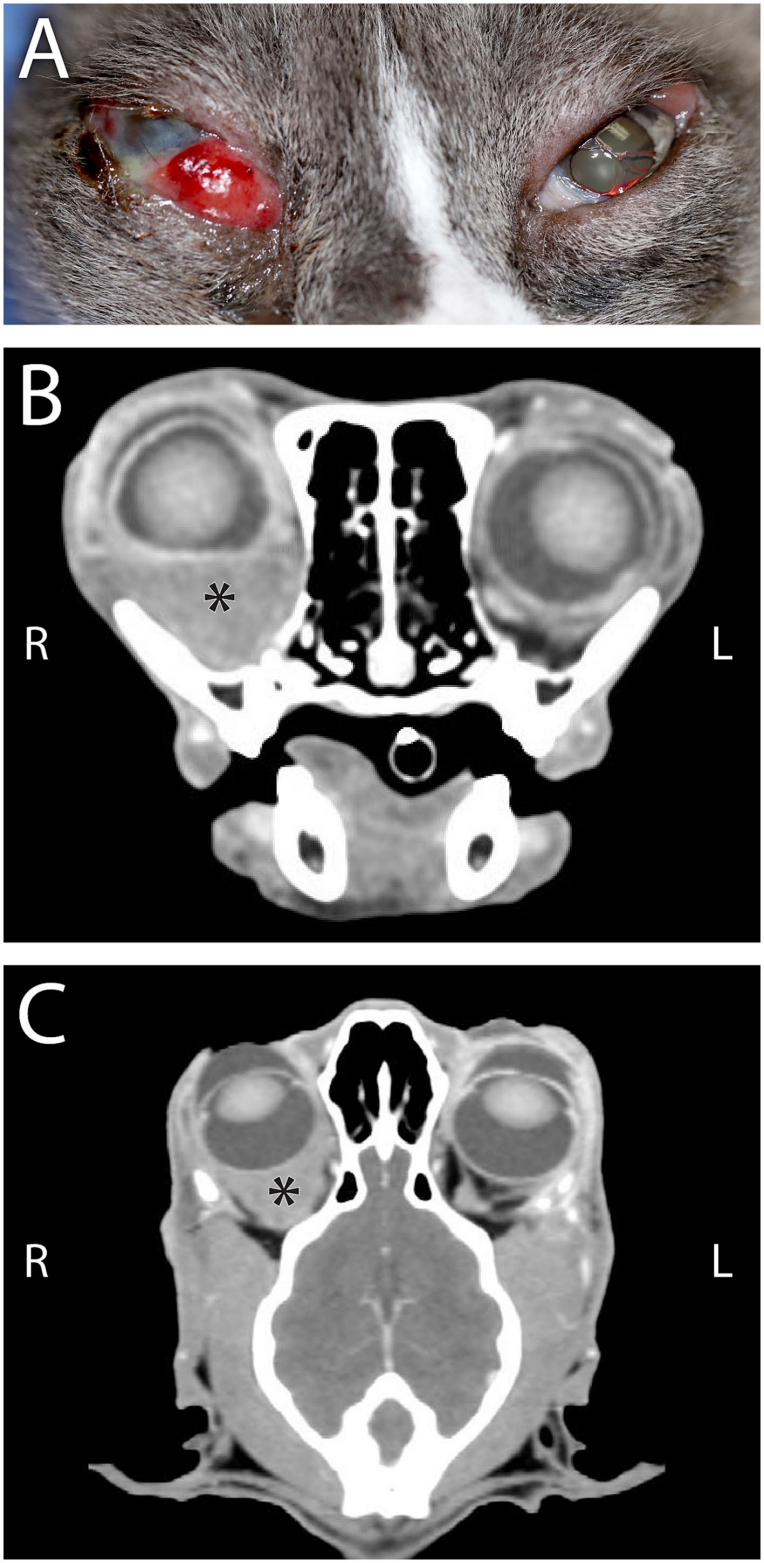
**(A)** A 16-year-old Siamese cat that was presented for suspected retrobulbar disease on the right side and a severely infected and deep corneal ulcer of the right eye (OD, i.e., ocular dexter). There is a moderately deep stromal ulcer and severe conjunctival swelling dorsolaterally on the left eye (OS, i.e., ocular sinister). **(B)** Computed tomography in transverse plane at the level of the maxillary fourth premolar teeth, in soft tissue algorithm post-contrast administration with window width (WW) 400 and window level (WL) 60, and **(C)** Computed tomography in dorsal/coronal plane at the approximate level of the tentorium cerebelli, in soft tissue algorithm post-contrast administration with WW/WL 400/60, revealed primary retrobulbar disease with homogeneously contrast enhancing soft tissue in the right retrobulbar regions (asterisk) with loss of definition of the normal fat and extraocular muscles with resultant moderate exophthalmia of the right globe, which is flattened ventrally. The sclera, iris, and ciliary body of the right globe were mildly thickened and contrast enhancing compared to the left. The mass was biopsied and diagnosed as disseminated large cell lymphoma (retrobulbar, splenic, and suspected pulmonary).

Of the 37 affected cats, 15 cats (40.5%) presented with primary retrobulbar disease. Out of these 15 cats, 9 cats were diagnosed with neoplasia (60%) (Figure 2). Two cases were reported as trauma or a foreign body in the retrobulbar space (13.3%). Two cases were reported as infectious (retrobulbar abscess) (13.3%). One case was reported as inflammatory due to neutrophilic conjunctivitis (6.7%). One case in the “other” category was diagnosed as a cyst (6.7%). Age of the cats that were reported with primary retrobulbar disease ranged from 3 months to 14 years, with a mean of 8.7 years. Cats with primary retrobulbar neoplasia ranged from 4.5 to 14 years, with a mean of 11.3 years. Cats with primary infectious/inflammatory retrobulbar disease ranged from 4.8 months to 13 years, with a mean of 3.6 years. The cat affected by a retrobulbar cyst was 3 months old.

There were 4 male castrated cats and 5 female spayed cats that were affected by primary neoplastic retrobulbar disease. There were 3 male castrated cats that were affected by primary inflammatory/infectious retrobulbar disease. The cat reported to have a cyst was male castrated. Breeds of the 9 cats with primary retrobulbar neoplasia included 6 domestic shorthair, 1 Siamese, 1 Maine Coon, and 1 domestic longhair. The 3 cats with primary infectious/inflammatory retrobulbar disease were domestic shorthair. The cat with a primary retrobulbar cyst was a domestic shorthair cat.

The most common type of neoplasia was lymphoma (*n* = 5, 55.6%) followed by squamous cell carcinoma (*n* = 2, 22.2%), adenocarcinoma (*n* = 1, 11.1%), and melanoma (*n* = 1, 11.1%).

Clinical signs as reported by the client are reported in Table 2. The most common clinical signs observed by the clinical service were ocular (*n* = 12, 80%) and respiratory (*n* = 4, 26.7%) that included ocular discharge (*n* = 9, 60%), decreased retropulsion (*n* = 7, 46.7%), decreased pupillary light reflex (*n* = 4, 26.7%), exophthalmia (*n* = 8, 53.3%), and nasal discharge (*n* = 4, 26.7%).

**Table 2:** Clinical signs, as reported by the client, that were present in cats diagnosed with primary retrobulbar disease, and the specialty service to which the cat was initially presented.

	Ocular signs	Respiratory signs	Oral signs	Ocular + respiratory	Ocular + oral	Oral + respiratory	Oral + neurologic signs	No clinical signs associated with retrobulbar disease
Ophthalmology	6			1				
Internal medicine		1					1	1
Medical oncology				1				
Dentistry and oral surgery	1							
Neurology	1							
Emergency								2

“Ocular signs” include exophthalmos, periorbital swelling, chemosis, blepharospasm, decreased pupillary light reflex, and/or ocular discharge, or strabismus. “Respiratory signs” include difficulty breathing, nasal discharge, epistaxis, sneezing, stertorous breathing, facial asymmetry, or open mouth breathing. “Oral signs” include difficulty and painful while eating, or difficulty opening the mouth. “Neurologic signs” include circling, ataxia, or pacing. If multiple different clinical signs were reported, a combination category was notated (i.e., ocular signs + respiratory signs).

Eight patients received fine needle aspirates and cytology. Biopsy and histopathology were performed in 6 cats. Culture and susceptibility were performed in 4 cats. Fungal culture was performed in 1 cat. Two of the patients that previously received cytology were euthanized and necropsy was performed. No biopsy or cytology was performed for the 2 patients with traumatic etiology. The 3 patients that were diagnosed with infectious/inflammatory disease were treated with medical management. Clinical signs subsequently resolved in 2 patients and the third patient was lost to follow up. The patient that was diagnosed with a foreign body in the retrobulbar space was treated with surgical removal and lost to follow up. The patient that had trauma causing primary retrobulbar disease is still alive at the time of writing of the manuscript, 6 years after the event. The patient who had a retrobulbar cyst underwent complete surgical resection of the affected tissue. Subsequent histopathological analysis revealed the tissue to be a cystic choristoma. Clinical signs resolved post-operatively. Two patients that were diagnosed with neoplasia were euthanized less than one month after diagnosis. One patient that was diagnosed with neoplasia was euthanized at the time of diagnosis. One patient who was diagnosed with large granular lymphoma of the left retrobulbar space received palliative chemotherapy which consisted of cyclophosphamide, doxorubicin, vinblastine, and prednisolone (CHOP therapy). This patient had a relapse halfway through treatment and also received L-asparaginase and cytarabine. Due to progression of disease, this cat was euthanized 3 months post-diagnosis. One patient with neoplasia was treated surgically via exenteration about 1.5 years before a CT scan was performed confirming its recurrence; this patient was euthanized about 6 months post-recurrence. One patient was originally diagnosed with high grade B cell lymphoma. The patient was administered one dose of L-asparaginase. After being on palliative care of methylprednisolone and buprenorphine with ocular medications (dorzolamide ophthalmic solution 2% and eye lubrication), clinical signs improved and patient survived 1 month after the contrast CT scan and was then euthanized. Three patients that were diagnosed with neoplasia were lost to follow up.

### 3.2 Secondary retrobulbar disease

**Figure 3:**
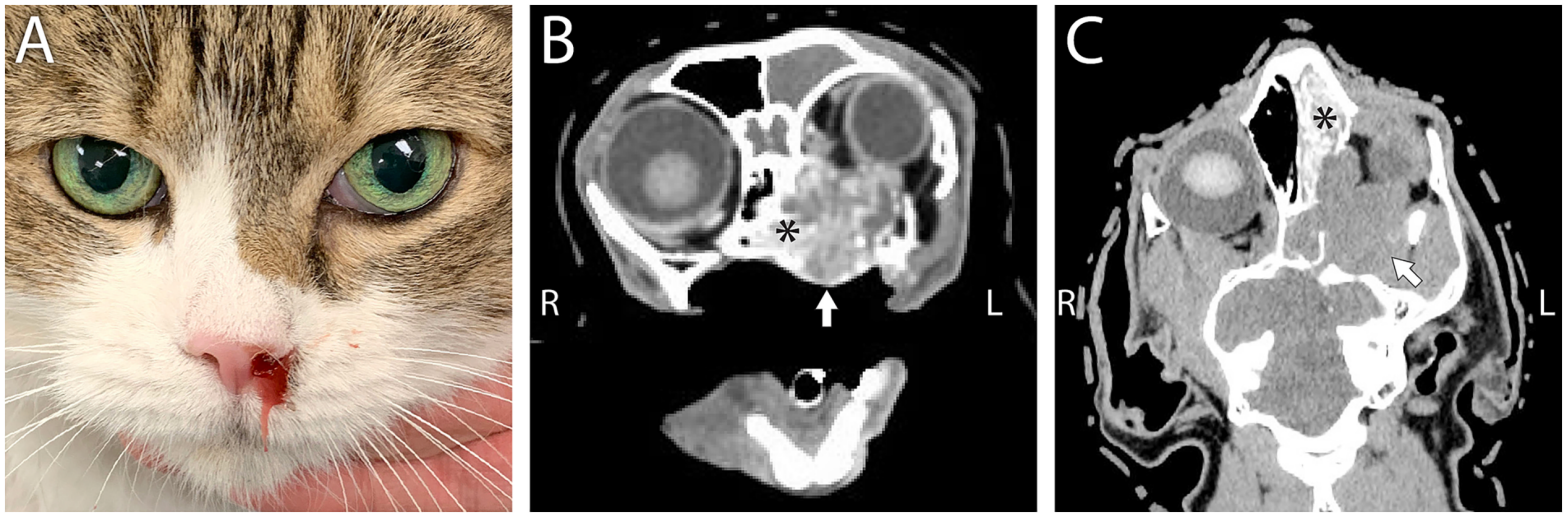
**(A)** A 12-year-old female spayed domestic medium hair that was presented due to left-sided bloody nasal discharge. Note the subtle third eyelid protrusion OS and prominence of the left globe compared to OD suggestive of mild exophthalmos. **(B)** Computed tomography in transverse plane at the level of the maxillary first molars, in soft tissue algorithm post-contrast administration with WW/WL 400/60 and **(C)** dorsal/coronal plane at the approximate level of the interthalamic adhesion, in bone algorithm post-contrast administration with WW/WL 400/60 revealed a large mass involving and obliterating the left nasal cavity (asterisk) and extending into the retrobulbar space (white arrows) consistent with secondary retrobulbar disease. There is slight intracalvarial extension as well as obstructive frontal sinusitis and exophthalmos. The mass was diagnosed as a poorly differentiated, intermediate to high grade, nasal carcinoma.

Out of the 22 cats reported to have secondary retrobulbar disease, 21 cats (95.5%) were diagnosed with neoplasia (Figure 3). One cat (4.5%) was reported to have infectious/inflammatory secondary retrobulbar disease (retrobulbar abscess).

**Figure 4:**
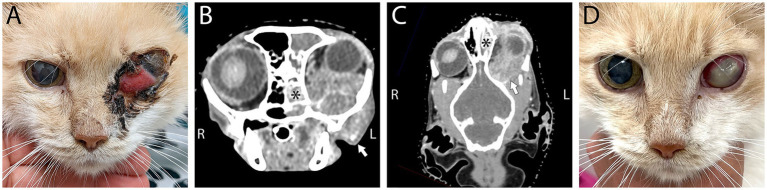
A 13-year-old domestic shorthair cat affected by left-sided retrobulbar disease **(A)**. Note the marked third eyelid protrusion, marked third eyelid conjunctival hyperemia, and ocular/periocular crusted discharge. The globe is not easily visible in this photo. The cat underwent conventional CT scan with and without contrast. **(B)** Computed tomography in transverse plane at the rostral aspect of the cribriform plate, in soft tissue algorithm post-contrast administration with WW/WL 400/60 and **(C)** Computed tomography in dorsal/coronal plane at the approximate level of the mesencephalic aqueduct, in soft tissue algorithm post-contrast administration with WW/WL 400/60 post-contrast administration demonstrating a large poorly defined, left retrobulbar mass (white arrows) with intranasal involvement (asterisk) and secondary exophthalmos. The mass was biopsied and diagnosed as high grade B-cell lymphoma. **(D)** Following 1-month of chemotherapy the majority of the swelling was reduced. Ophthalmic examination at that time revealed a stromal corneal ulcer, keratitis, and a cataract that rendered the eye non-visual.

**Figure 5:**
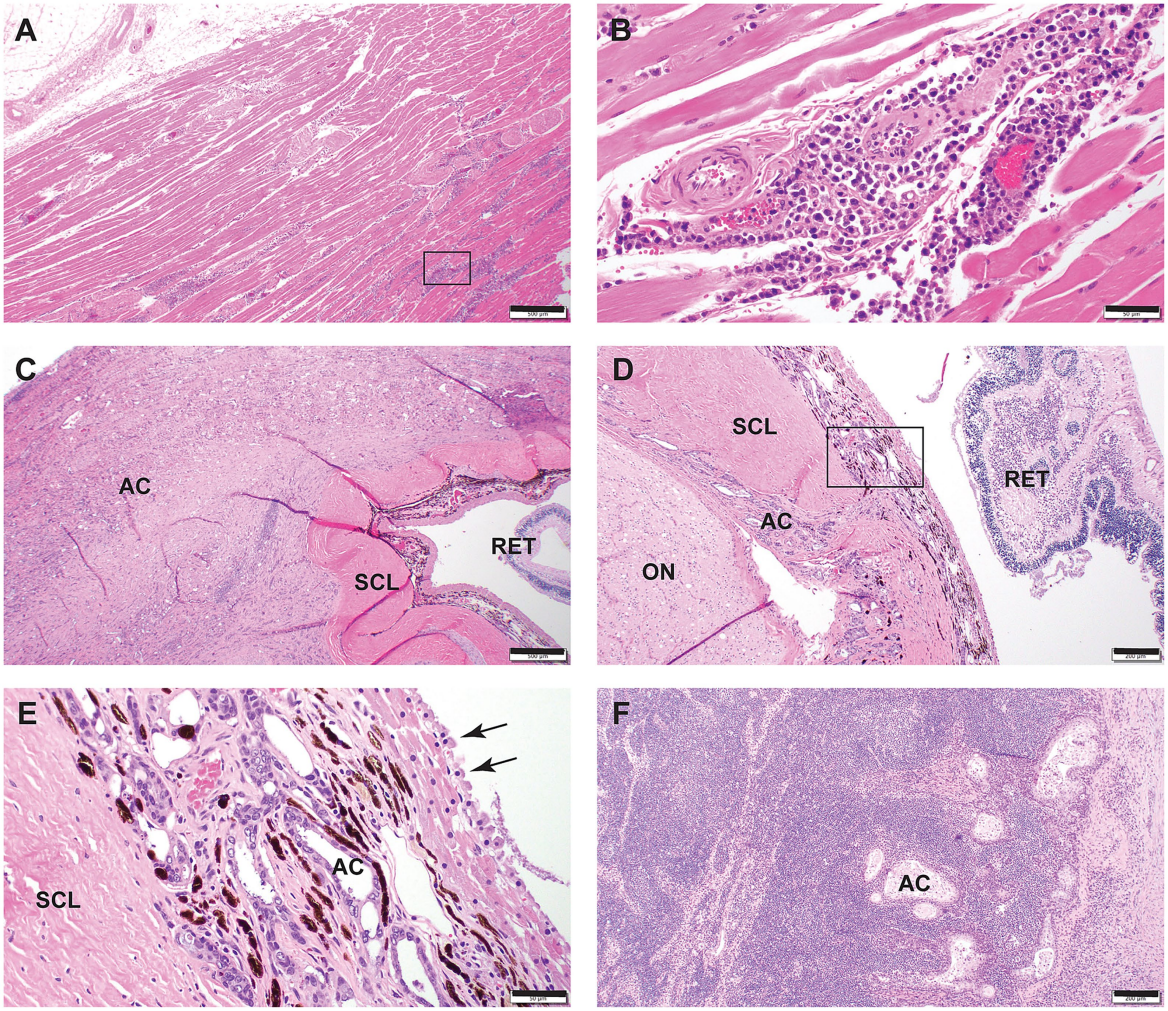
Histopathologic sections from selected cases of retrobulbar lymphoma in a 15-year-old cat **(A,B)** and adenocarcinoma in a 4-year-old cat **(C–F)** that underwent necropsy following euthanasia due to retrobulbar neoplasia. **(A)** Low magnification of retrobulbar muscle and adipose tissue. Note, in the lower right corner of the photomicrograph, that the myofibers are separated by densely cellular infiltrate. Hematoxylin and eosin staining. Bar = 500 μm. **(B)** High magnification of the rectangular area in capture A. Note that the perivascular space is expanded by monomorphic round cell infiltrate that was determined to be large granular T cell lymphoma with ancillary diagnostics. Hematoxylin and eosin staining. Bar = 50 μm. **(C)** Low magnification of the retrobulbar space. The sclera (SCL) is tightly engulphed by a densely cellular adenocarcinoma (AC). The retina (RET) is detached. Hematoxylin and eosin staining. Bar = 500 μm. **(D)** Additional retrobulbar segment demonstrating adenocarcinoma invasion through the sclera into the uvea. The neoplastic growth of the adenocarcinoma (AC) dissects between the sclera (SCL) and the optic nerve (ON). The retina (RET) is detached. Hematoxylin and eosin staining. Bar = 200 μm. **(E)** High magnification of the area enclosed in the rectangle in D. Adenocarcinoma cells infiltrate the uvea which forms poorly defined ducts and acini. Overlaying the infiltrated uvea is the tapetum lucidum and retinal pigmented epithelium undergoing cell rounding /tombstoning (black arrows), indicating a true, clinical retinal detachment. Hematoxylin and eosin staining. Bar = 50 μm. **(F)** Mandibular lymph node from the patient shown in C–E. Note adenocarcinoma cells forming ductular structures in the substance of the lymph node. Hematoxylin and eosin staining. Bar = 200 μm.

The most common neoplasia reported was lymphoma (*n* = 5, 24%) (Figures 4, 5A,B), followed by squamous cell carcinoma (*n* = 4, 19%), adenocarcinoma (*n* = 4, 19%) (Figures 5C–F) and an undifferentiated sarcoma (*n* = 3, 14%). There was 1 case each of a peripheral nerve sheath tumor, fibrosarcoma, undifferentiated carcinoma, feline restrictive orbital myofibroblastic sarcoma, and osteosarcoma.

The age of the cats that were reported at the time the CT scan was performed was 8 to 18 years old with a mean of 13 years. The age of the cats with secondary neoplastic retrobulbar disease ranged from 5 to18 years with a mean age of 10.7 years. The age of the cat with secondary infectious/inflammatory disease was 8 years. The weight of cats with secondary neoplastic disease ranged from 3 kg to 7 kg, with a mean of 5 kg. The weight of cat with secondary infectious/inflammatory retrobulbar disease was 5 kg. The sex of cats with secondary neoplastic retrobulbar disease included 13 female spayed cats, 7 male castrated cats, and 1 female intact cat. The cat with infectious/inflammatory disease was a female spayed cat.

Breeds of the 21 cats with secondary retrobulbar neoplasia included 12 domestic shorthair, 4 domestic longhaired cats, 3 domestic medium hair, 1 Maine Coon, and 1 Bengal. The breed of the cat that had secondary infectious/inflammatory retrobulbar disease was a domestic shorthair.

Clinical signs reported by the client are reported in Table 3. The most common clinical signs observed by the client were respiratory signs including nasal discharge, sneezing, and stertorous breathing. The most common clinical signs observed by the clinical service were ocular (*n* = 14, 63.6%) and respiratory (*n* = 15, 68.1%) that included nasal discharge (*n* = 10, 45.4%), and stertorous respiration (*n* = 4, 18.2%), ocular discharge (*n* = 10, 45.5%), decreased retropulsion (*n* = 8, 36.3%), decreased pupillary light reflex (*n* = 7, 31.8%), and exophthalmia (*n* = 4, 18.2%).

**Table 3:** Clinical signs, as reported by the client, that were present in cats diagnosed with secondary retrobulbar disease, and the specialty service to which the cat was initially presented.

	Ocular signs	Respiratory signs	Oral signs	Ocular + respiratory	Oral + respiratory	Ocular + Oral + Neurologic Signs	No clinical signs associated with retrobulbar disease
Ophthalmology	3	1					
Internal medicine	1	5		1			
Medical oncology	1	2		1			
Radiation oncology		1	1		2	1	
Dentistry and oral surgery	1						
Soft tissue surgery							1

“Ocular signs” include exophthalmos, periorbital swelling, chemosis, blepharospasm, decreased pupillary light reflex, and/or ocular discharge, or strabismus. “Respiratory signs” include difficulty breathing, nasal discharge, epistaxis, sneezing, stertorous breathing, facial asymmetry, or open mouth breathing. “Oral signs” include difficulty and/or painful while eating, or difficulty opening the mouth. “Neurologic signs” include circling, ataxia, or pacing. If multiple different clinical signs wer reported, a combination category was notated (i.e., ocular signs + respiratory signs).

Biopsy and histopathology were performed in 16 cats. Fine needle aspirate and cytology were performed in 5 cats and were all of diagnostic quality. Fungal culture was performed in 3 cats. Culture and susceptibility were performed in 2 cats. One cat was diagnosed with secondary neoplasia during necropsy. One cat that had a biopsy and histopathology performed was euthanized and necropsy was performed. Another cat that was diagnosed with neoplasia by fine needle aspirate and cytology also received necropsy after euthanasia. Biopsy and histopathology were considered conclusive in 15/16 cats (93.6%). Of the 2 cats that received culture and susceptibility, only 1 result was consistent with infection (50%). All 3 cats that received fungal culture were not ultimately diagnosed with a fungal etiology.

The most common diagnosis for cats with secondary retrobulbar disease was a primary nasal tumor (*n* = 15, 71.4%). Of these, 5 cases were diagnosed as lymphoma (23.8%), 3 were diagnosed as undifferentiated sarcoma (19%), 3 were adenocarcinomas (14.3%), 2 were squamous cell carcinoma (9.5%), 1 feline restrictive orbital myofibroblastic sarcoma (4.8%), and 1 osteosarcoma (4.8%). Other sites of neoplastic origin included the mandible (*n* = 2, 9.5%),the calvarium (*n* = 2. 9.5%), and the eyelid (*n* = 2, 9.5%). Of the 2 mandibular neoplastic cases, 1 was diagnosed as a squamous cell carcinoma (50%) and 1 undifferentiated mandibular carcinoma (50%). Of the calvarial neoplastic cases, 1 was a squamous cell carcinoma (50%) and 1 adenocarcinoma (50%). Of the 2 eyelid neoplastic cases, 1 was a fibrosarcoma (50%) and 1 peripheral nerve sheath tumor (50%). The cat that had infectious/inflammatory disease was diagnosed with neutrophilic, lymphoplasmacytic rhinitis.

Six cats underwent radiation treatment, and 1 was euthanized less than one month after diagnosis. Out of these 6 patients, 2 cats received surgery (orbital exenteration) and then received follow-up palliative radiation treatment. Of these two cats, one cat had completed treatment, but due to recurrence, was euthanized 6.5 months post-diagnosis (186 days). The other cat received palliative radiation therapy after orbital exenteration for a peripheral nerve sheath tumor and was euthanized 11 months post-diagnosis (329 days). One cat that received palliative radiation therapy for an oral squamous cell carcinoma completed treatment, but was euthanized 9.7 months post-diagnosis (292 days). The last two cats were lost to follow up after completion of radiation therapy (one received palliative and the other received full course of radiation therapy). One cat underwent chemotherapy for nasal lymphoma and was euthanized 1 month after diagnosis. Two cats received surgery (exenteration) for the affected eye. Of these two cats, one was euthanized at an unknown date, and the other was lost to follow up. Seven cats received palliative care. One cat that was diagnosed with infectious secondary retrobulbar disease was euthanized 7.5 months (224 days) after diagnosis due to progressive clinical signs despite medical management with antibiotics. Overall, 3 cats with secondary neoplastic retrobulbar disease were euthanized at the time of diagnosis, 2 cats were euthanized less than 1 month after diagnosis, 2 cats (one from neoplasia and the other from an infectious disease process) were euthanized 2–3 months after diagnosis, 1 cat was euthanized 6.5 months after diagnosis, 2 cat was euthanized 9 months after diagnosis,1 cat was euthanized 11 months after diagnosis, and 8 cats overall were lost to follow up. Two additional cats were reported to be euthanized, but the exact date was not recorded.

### 3.3 CT findings

The CT findings are summarized by disease category in Table 4. Orbital osteolysis (*p* < 0.0042) and reduction of retrobulbar fat (*p* < 0.0164) were significantly associated with neoplasia. Other characteristics including osseous distortion, periosteal reaction, zygoma distortion, zygoma osteolysis, zygoma periosteal reaction, salivary gland enlargement and displacement, retrobulbar mass effect, exophthalmos, enophthalmos, misshapen globe, and mandibular or retropharyngeal lymph node enlargement did not significantly differ between neoplastic and infectious/inflammatory disease processes. The presence of a retrobulbar mass was more commonly associated with neoplasia, but did not reach significance (*p* = 0.0723).

**Table 4:** Computed tomographic (CT) findings associated with retrobulbar disease in 37 cats.

CT findings	Number affected: unilateral, bilateral		
	Infectious/inflammatory (*n* = 4)	Neoplasia (*n* = 30)	Trauma/other (*n* = 3)
Orbit: osseous distortion	1, 0	8, 1	2, 0
Orbit: osteolysis	1, 0	**20, 3**	0, 0
Orbit: periosteal reaction	0, 0	10, 2	0, 0
Zygomatic arch: osseous distortion	0, 0	4, 0	1, 0
Zygomatic arch: osteolysis	0, 0	4, 0	0, 0
Zygomatic arch: periosteal reaction	0, 0	8, 0	0, 0
Zygomatic salivary gland enlargement	1, 0	3, 0	0, 0
Zygomatic salivary gland compression/displacement	1, 0	16, 1	1, 0
Reduction of retrobulbar fat	4, 0	**27, 2**	0, 0
Retrobulbar mass effect	4, 0	22, 2	1, 0
Retrobulbar mass	1, 0	19, 2	1, 0
Exophthalmos*	4, 0	17, 1	1, 0
Enophthalmos*	0, 0	2, 0	0, 0
Misshapen globe*	2, 0	13, 2	1, 0
Mandibular lymph node enlargement	4, 0	11, 2	0, 0
Medial retropharyngeal lymph node enlargement	4, 0	15, 4	0, 0

The total number of cats included in each category are given at the top of each column. The value on the left signifies the total number of cases affected with unilateral retrobulbar disease and the number on the right signifies the total number of cases with bilateral retrobulbar disease. Values in bold indicate a statistically significant difference between the infectious/inflammatory and neoplastic disease.

*Exenteration was previously performed in one cat, which could not be included in the statistical analysis.

Sensitivity and specificity of each characteristic are summarized in Table 5. The presence of orbital osteolysis overall had a moderate specificity and sensitivity for neoplasia. The presence of decreased retrobulbar fat had a high sensitivity but overall low specificity for neoplasia.

**Table 5:** Sensitivity and specificity of individual CT findings for diagnosis of neoplasia in cats.

CT Finding	Neoplasia	
	Sensitivity	Specificity
Orbit: osteolysis	77% (59–88%)	86% (49–99%)
Decreased retrobulbar fat	97% (83–100%)	43% (16–75%)

Values in parentheses represent the 95% confidence levels.

Mass contrast enhancement for every case was recorded. A heterogenous contrast enhancement was observed in 11 neoplastic cases (29.7%). A homogenous contrast enhancement was observed in 16 cases (43.2%). There were no inflammatory/infectious disease cases that exhibited heterogenous or homogenous contrast enhancement. There were 4 infectious/inflammatory disease cases (10.8%), 2 neoplastic cases (5.4%), and 1 other case (cyst) (2.7%), that had peripheral contrast enhancement. There was 1 neoplastic case (2.7%) and the 2 previously diagnosed trauma cases (5.4%) that did not have any contrast enhancement.

### 3.4 Survival and outcome

**Figure 6:**
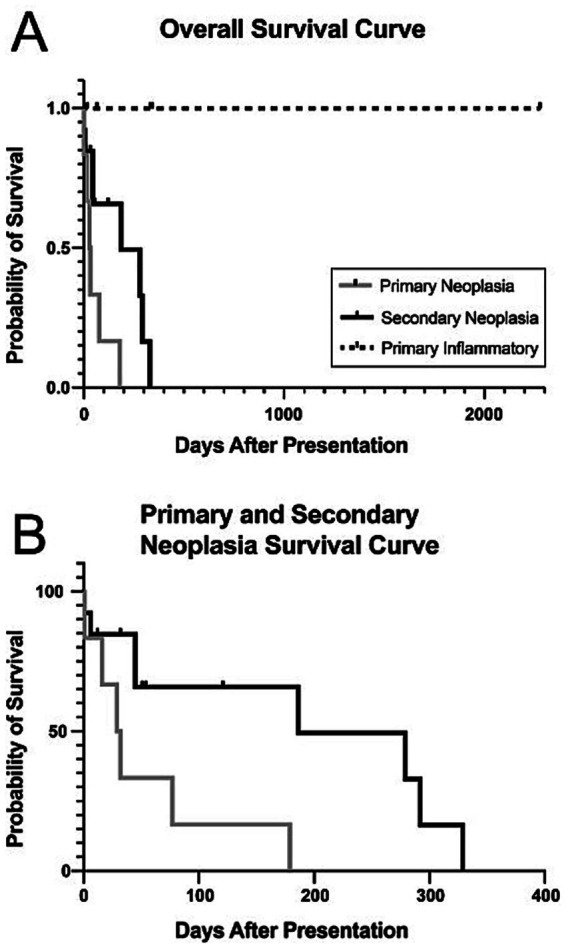
Kaplan–Meier survival curve in days for 36 cats affected with primary and secondary retrobulbar disease. The one cat diagnosed with secondary infectious disease was not included in this data set.

The survival data results were collected from cats that presented with primary neoplastic retrobulbar disease, primary inflammatory/infectious disease, and secondary neoplastic retrobulbar disease. (Figure 6). The one cat that was diagnosed with secondary infectious disease was not included in this data set.

Three of the 9 cats with primary neoplastic retrobulbar disease were lost to follow up. The range of survival days for the 6 patients with primary neoplasia that were euthanized was 1–179 days, with a median survival time of 30.5 days. The cat that had a survival time of 179 days had a melanoma of the right conjunctiva with recurrence after exenteration. Ten of the 21 cats with secondary neoplastic retrobulbar disease were lost to follow up. The range of survival days of the remaining 11 patients was 0–329 days. The median survival time was 186 days. Cats that were presented with primary neoplastic retrobulbar disease had a significant difference in survival outcome compared to cats that presented with secondary neoplastic retrobulbar disease (*p* < 0.0131). The median survival time was not reached for cats diagnosed with primary infectious/inflammatory disease as all were living at the time of last follow-up. All cats had resolved clinical signs, except for one which was lost to follow up. The cats that were presented with an infectious/inflammatory primary retrobulbar disease had a significant difference in survival outcome compared to the cats that had both primary and secondary neoplastic retrobulbar disease (*p* < 0.0004).

## 4 Discussion

This retrospective study described the clinical features and CT findings of cats affected with retrobulbar disease. We demonstrated several clinically important features of retrobulbar disease. First, neoplastic lesions were more common than infectious/inflammatory diseases in both primary and secondary retrobulbar disease groups as hypothesized. However, infectious/inflammatory disease remained common and typically carried favorable prognosis. We also demonstrated that orbital osteolysis and reduction of retrobulbar fat were significantly associated with neoplasia. Furthermore, the presence of orbital osteolysis overall had a moderate correlation specificity and sensitivity with neoplasia. Finally, infectious/inflammatory retrobulbar disease treated medically and surgically had generally favorable outcome.

Clinical identification of retrobulbar disease in cats may be subtle and challenging to determine solely based on the history, owner observations, and physical examination findings. Previous studies reported that decreased ocular retropulsion was consistent with retrobulbar disease in dogs (1, 3, 21). However, only 13 cats out of the 37 with primary and secondary retrobulbar disease (35%) presented with ocular signs. These findings were more consistent with a previous study where only 30% of the cases had decreased retropulsion (2). The most common ocular clinical sign of both primary and secondary retrobulbar disease was ocular discharge (19/37, 51.3%) which is slightly more frequently noted than in a previous report where 41% (15/37) of cases with retrobulbar neoplasia had ocular discharge (2). The other common ocular clinical signs were exophthalmia, decreased retropulsion, and decreased pupillary light reflex. In contrast, one case that had a foreign body did not exhibit ocular clinical signs. Hence, we conclude that there is no specific or sensitive clinical sign that would be exclusively indicative of retrobulbar disease.

The most common clinical signs observed by owners associated with primary retrobulbar disease were ocular signs such as exophthalmos and ocular discharge; whereas in secondary retrobulbar disease, respiratory signs were the most common presenting complaint. We noted that patients were presented to the specialty service due to the most obvious clinical signs to the owner. These findings are consistent with a previous study and respiratory signs, typically due to nasal neoplasia, were more common as compared to ocular signs (2). The majority of the patients that presented to either the medical oncology and radiation oncology services already had a definitive diagnosis with either fine needle aspirate and cytology or biopsy and histopathology performed at their primary veterinarian.

We demonstrated that orbital osteolysis and reduction of retrobulbar fat were significantly associated with neoplasia and that the presence of orbital osteolysis overall had a moderate specificity and sensitivity in detecting neoplasia. This finding agrees with a previous study where abnormalities with the orbital bones was more predictive of orbital neoplasia; however, the previous study did not specify about specific changes to the orbital bones (31). Due to the complexity of evaluating the retrobulbar space, advanced diagnostic imaging that identifies both bone and soft tissue structures is important to determine the nature and extent of retrobulbar diseases. In the present study, contrast CT was essential at determining the origin and evaluating the extent of retrobulbar disease and the structures involved. In our previous study in dogs, the CT findings that were significantly associated with neoplasia were: orbital osteolysis, orbital periosteal reaction, and the presence of a retrobulbar mass (1). The CT findings that were significantly associated with infectious/inflammatory retrobulbar disease were: zygomatic salivary gland enlargement, retrobulbar mass effect, and mandibular lymph node enlargement (1). In the current study, we discovered that there was no specific CT finding that was consistent with infectious/inflammatory primary or secondary retrobulbar disease in cats.

Although CT findings are essential in guiding diagnosis, a definitive diagnosis obtained by histopathology, cytology, or culture and susceptibility is essential in determining the underlying pathogenesis and formulating targeted therapy. However, sampling the retrobulbar area is challenging and carries risk of damage to the eye and neurovascular structures. A previous study reported that 5 out of 7 cats (71%) with confirmed orbital abscess had confirmed positive culture results (14). In addition, in another study with 21 cats with orbital neoplasia, 5 of 21 cases were diagnosed with fine needle aspirate of the retrobulbar or external masses (24%), but 6 of 21 of samples were not diagnostic. Neoplasia was confirmed in 16 of 21 (76%) cases with surgical biopsy or tissue (i.e., necropsy) obtained (76%) (5). Furthermore, our group demonstrated that in retrobulbar diseases in dogs, the histopathology and cytology results are not always consistent with each other and that obtaining biopsy for histopathology is recommended, when possible, to increase the chances of achieving a definitive diagnosis (1).

In this report, the majority of retrobulbar cases was secondary rather than primary, and the majority of cases was diagnosed as a neoplastic process. Among these cases, nasal lymphoma was the most common neoplasia diagnosed, which is consistent with previous studies (2, 11). This also is agreement with another study where 9 of 11 cases affected with nasal disease had extension into the retrobulbar space and were neoplastic (10). This study supports other studies that also revealed that the cases diagnosed with primary retrobulbar disease were mostly affected by neoplasia (60%). However, we demonstrated that infectious/inflammatory diseases or foreign bodies in the retrobulbar space of cats are also common (40%) and carried a significantly favorable outcome. Out of the six cases, five cats had resolved clinical signs with either medical or surgical management and one was lost to follow-up. This agrees with past reports that demonstrated that infectious/inflammatory diseases in the retrobulbar space treated medically or surgically typically carried a good prognosis (13–15). There were no fungal infection cases in this retrospective study, which may reflect a location bias as fungal diseases are rare in California. However, those cases previously reported favored a poor or guarded prognosis as surgical intervention (exenteration) and follow up medical management with antifungal medications is needed (16–19). As described in these case reports, there are risks of systemic side effects to the anti-fungal medications such as neurologic complications and kidney disease. No parasitic cases were reported in this study; however, there have been reports of “Onchocerca lupi” causing ocular disease that is treatable and carries a good prognosis (20).

A limitation of this study is that the cats that were included in this study were from a tertiary referral center. Also, the inclusion criteria required contrast CT and either histopathology or cytology to be performed. Therefore, this may not represent the general population of retrobulbar disease in cats. Another limitation of this study is that treatment procedures were not standardized; treatments recommended to each patient were determined based on a variety of specialty clinicians. There is also a chance that some retrobulbar disease processes were not as complex and did not warrant a referral to a specialist. Dental disease causing orbital disease has been reported in dogs and cats; however, the majority of these cases may be treated with dental extractions or antibiotics at the primary clinician (21, 32). Also, the preference of imaging modality is dependent on the receiving specialty service and clinician, and this study only utilized contrast CT findings rather than include MRI or ocular ultrasound. There are also cases where cone beam CT or skull radiographs were performed that were not included in this study as these two imaging modalities are not appropriate and not reliable in determining soft tissue involvement (33, 34).

In conclusion, the retrobulbar space is a complex anatomical region and disorders in this area may be challenging to diagnose and treat. It is difficult to distinguish other ocular or orbital disease processes solely based on the initial presenting complaints or physical exam findings, and there is a clear need for advanced imaging. CT has been confirmed to be an essential part of diagnosis and characterization of the extent of retrobulbar disease in cats, as it is in the dog; however, further diagnostics such as histopathology, cytology, bacterial culture and susceptibility, or fungal cultures are needed to further support and guide treatment options. Furthermore, cats that present with primary infectious/inflammatory retrobulbar disease carry favorable prognosis with either medical or surgical intervention compared to those cats that have primary or secondary neoplastic retrobulbar disease.

## Data Availability

The raw data supporting the conclusions of this article will be made available by the authors, without undue reservation.
